# CD11c^+^ microglia: From basic research to clinical application

**DOI:** 10.4103/NRR.NRR-D-25-00868

**Published:** 2025-12-30

**Authors:** Zipeng Zhou, Yongfei Zhao, Xiangyi Fan, Jinhui Zhang, Ruihan Niu, Yifei Ma, Fei Xie, Peifu Tang, Xifan Mei, Licheng Zhang, Junhao Deng

**Affiliations:** 1 Department of Orthopedics, Senior Department of Orthopedics, Chinese PLA Hospital, Beijing, China; 2 Department of Otolaryngology-Head and Neck Surgery, First Affiliated Hospital of Jinzhou Medical University, Jinzhou, Liaoning Province, China; 3 Liaoning Vocational College of Medicine, Shenyang, Liaoning Province, China; 4 School of Life Science, Tsinghua University, Beijing, China

**Keywords:** CD11c^+^ microglia, disease-associated microglia, microglial subsets, myelination, neurodegeneration, neuroimmunology, neuroinflammation, neurological diseases, phagocytosis

## Abstract

CD11c^+^ microglia are a functionally specialized subpopulation of microglia that play a crucial role in the pathophysiological processes of various central nervous system diseases. This review synthesizes compelling evidence that CD11c^+^ microglia exhibit unique transcriptomic and phagocytic characteristics. These characteristics distinguish them from homeostatic microglia and support their specialized functions. During development, CD11c^+^ microglia are crucial for the maturation of oligodendrocytes and the integrity of white matter, particularly in regions such as the corpus callosum and cerebellum. In preclinical models of neurodegenerative diseases (such as Alzheimer’s disease and amyotrophic lateral sclerosis) and central nervous system injuries (such as stroke and spinal cord injury), they are consistently associated with neuroprotective phenotypes. CD11c^+^ microglia exhibit enhanced phagocytic capacity near amyloid plaques and damaged neurons, helping to clear pathological protein aggregates and cell debris, thereby reducing neurotoxicity and promoting a repair environment. The current consensus is that specific microenvironmental cues, particularly hazard signaling molecules damage-associated molecular patterns and cytokines (such as interferon-γ), are the main drivers of the differentiation and activation of CD11c^+^ microglia. Among these, the TREM2-APOE signaling axis is a key and widely accepted regulatory pathway for their survival, proliferation, and functional status. The plasticity of CD11c^+^ microglia is regulated by multiple signaling pathways, including CSF1R, SIRPα-CD47, interferon-γ, and the complement cascade. Emerging therapeutic strategies aim to regulate their activities through gene targeting, metabolic intervention, and immune regulation using TREM2 agonists, CSF1R inhibitors, or nanopharmacological methods. However, challenges remain in defining specific CD11c^+^ biomarkers, understanding environment-dependent functions, and achieving targeted delivery. Future prospects depend on clearly addressing individual developmental issues, deciphering the molecular switches that control phenotypic plasticity, and developing highly specific therapeutic strategies to leverage their beneficial functions, thereby paving the way for new intervention methods for neurological diseases.

**Facts**

• The presence of CD11c^+^ microglia is dynamic, occurring during development, aging, and disease states, and is closely related to Alzheimer’s disease, Parkinson’s disease, multiple sclerosis, stroke, and spinal cord injury.

• CD11c^+^ microglia have dual functions: under certain conditions, they can promote neuroprotection and repair, but they may also be involved in chronic neuroinflammation and disease progression.

• Targeting CD11c^+^ microglia by regulating their phenotypic transformation, immune activity, and neuroprotective functions may become a new approach for treating neurodegenerative diseases.

**Open questions**

• CD11c^+^ microglia exhibit region-specific functional heterogeneity in the central nervous system (e.g., in the corpus callosum, cerebellum, and hippocampus). How does the local microenvironment shape their transcriptomic and phagocytic characteristics during development and disease progression?

• What are the precise molecular mechanisms that determine the phenotypic plasticity of CD11c^+^ microglia in different disease environments?

• Is it possible to achieve *in vivo* specific targeting of CD11c^+^ microglia without affecting homeostatic microglia? Which strategies are most promising for achieving spatial and temporal precision in therapeutic interventions?

## Introduction

Neural injury, repair, and regeneration constitute the core challenges in the field of neuroscience. Microglia, the resident immune cells of the central nervous system (CNS), are indispensable for maintaining CNS homeostasis and responding to pathological challenges (Paolicelli et al., 2022). Beyond their primary role as the brain’s immune sentinels, microglia participate in neural development, the phagocytosis of apoptotic cells, neuronal homeostasis, synaptic pruning, and neural circuit formation (Paolicelli et al., 2022). Their functionality varies across regions and evolves dynamically during brain maturation.

Among the various microglial subpopulations, CD11c^+^ microglia represent a critical subset of microglial cells in the CNS, characterized by the expression of CD11c (also termed integrin αX, encoded by Itgax). This transmembrane receptor participates in diverse cellular processes, including adhesion (Hou et al., 2025), migration (Ghena et al., 2025), antigen presentation (Singh et al., 2024), proangiogenic activities (Droho et al., 2023), inflammatory responses (Chauhan et al., 2024), and complement-dependent phagocytosis (Yousefpour et al., 2025). These cells exhibit functional significance across CNS development (Mayo et al., 2024; Ghena et al., 2025), homeostasis (Levack et al., 2022), and pathological conditions (Hu et al., 2025). Thus, understanding the mechanisms of CD11c^+^ microglia is pivotal for neuroimmune-targeted therapeutics.

Microglia originate from the blood islands of the yolk sac in the early stage of embryonic development and migrate to the brain around 4–5 weeks of development (Benmamar-Badel et al., 2020). They differentiate into mature branching forms by the 12^th^ week. These cells are maintained through self-renewal and are typically not replaced by peripheral progenitors under normal conditions. The self-sustenance of microglia depends on the colony-stimulating factor 1 receptor (CSF1R), which supports their independent maintenance in the CNS and distinguishes them from other macrophages (Qin et al., 2023).

Under homeostasis, microglia exhibit highly branched morphology (“resting”) for dynamic CNS surveillance. Upon pathological challenge (e.g., inflammation and trauma), they activate into enlarged, rounded, or amoeboid forms, with morphology directly correlating to injury severity (Woodburn et al., 2021).

For a long time in the past, the activation of microglia was simply divided into pro-inflammatory (M1) and anti-inflammatory (M2) states (Guo et al., 2022; Qin et al., 2023). They play an active role in immune defense. However, the polarization patterns of M1 and M2 are considered to simplify data interpretation when the individual development and functional significance of microglia have not yet been determined. This hinders the research progress and should be discarded. Microglia of different phenotypes exhibit distinct functions, which not only depend on their environment but also on their intrinsic characteristics (Hammond et al., 2019). Applications such as two-photon imaging, whole-genome transcriptomics and epigenomic analysis confirm this high plasticity (Wang et al., 2023).

Hammond et al. (2019) analyzed the RNA expression patterns of over 76,000 microglia in mice during development, old age, and after brain injury using high-throughput single-cell transcriptomics. They found that microglia were most diverse during early development and became less heterogeneous in adulthood, until perturbed by injury or aging. They identified distinct microglial states. These distinct microglial signatures can be used to better understand microglial function and to identify and manipulate specific subpopulations in health and disease.

CD11c^+^ microglia are a hallmark of disease-associated microglia (DAM) in Alzheimer’s disease (AD), characterized by Itgax upregulation (Benmamar-Badel et al., 2020) and enrichment around amyloid-β (Aβ) plaques (Tsering et al., 2025). This subset proliferates in multiple sclerosis (Magliozzi et al., 2025) and neuropathic pain (Tsuda and Kohno, 2023), making it a spatiotemporal marker for CNS pathology (Lopes et al., 2024). Their roles have been well-documented in early development and pathological states (Nomaki et al., 2024). As a special subpopulation with key immunomodulatory functions, their roles during embryonic development and under pathological conditions have been confirmed by multiple studies (Jia et al., 2023; Nomaki et al., 2024; Ghena et al., 2025).

CD11c^+^ microglia show significant spatial heterogeneity in different regions of the CNS (Nomaki et al., 2024). They are shared markers among multiple developmentally and pathologically relevant microglial subsets, such as axon tract-associated microglia (ATM) (Hammond et al., 2019), proliferative region-associated microglia (PAM) (Li et al., 2019), youth-associated microglia (YAM) (Silvin et al., 2022), and Arginase-1^+^ microglia (Stratoulias et al., 2023). These populations commonly exhibit high Itgax expression, suggesting conserved functional modules or regulatory mechanisms.

However, there are still contradictions and ambiguities regarding the origin of CD11c^+^ microglia and their specific functions in different pathological stages. Additionally, although CD11c is a common marker for multiple important functional subpopulations, its specificity as a surface marker is insufficient, and the lack of more refined molecular definitions severely restricts targeted research on these cells.

Therefore, this review aims to systematically organize and integrate the research progress of CD11c^+^ microglia in various neurological diseases. The core objective is to analyze in depth its functional mechanisms, regulatory pathways, and pivotal role in neural injury and repair.

## Search Strategy

To comprehensively collect literature related to CD11c^+^ microglia and neurological diseases, a rigorous search strategy was implemented. This review conducted a literature search using PubMed (https://www.ncbi.nlm.nih.gov/pubmed), covering research published from its inception to June 2025. Our search string combined keywords, synonyms, and Medical Subject Headings (MeSH), connected with Boolean operators (AND, OR, NOT) and wildcards (*) to capture spelling variations or plurals.

Specific inclusion criteria were applied: (1) study populations limited to human or animal models (e.g., mice and rats); (2) study types restricted to original research articles, reviews, or cohort studies; and (3) direct investigation of CD11c^+^ microglia in neurological diseases such as Alzheimer’s disease, Parkinson’s disease, multiple sclerosis, neuropathic pain, traumatic brain injury, or spinal cord injury.

Exclusion criteria include: (1) studies not related to CD11c^+^ microglia, (2) studies conducted in non-disease contexts (e.g., basic microglial biology), (3) non-peer-reviewed content (e.g., conference abstracts, letters), and (4) duplicate or incomplete data reports. After the initial title and abstract screening, full-text reviews were conducted to ensure alignment with the predefined criteria, ensuring methodological transparency and reproducibility. The final number of articles included in the review analysis was 194, of which 143 were classic articles published in the past 5 years.

## Discovery and Identification of CD11c^+^ Microglia

CD11c has traditionally been regarded as a marker molecule of dendritic cells (DCs), and is mainly highly expressed in mouse conventional dendritic cells (cDCs) and plasmacytoid dendritic cells (pDCs). It may also occur in specific lymphocyte subsets (Schittenhelm et al., 2017). Interestingly, a human study has further shown that its expression profile is more extensive. In addition to DCs, CD11c can also be detected in monocytes, macrophages, granulocytes, and natural killer cells in human (Schittenhelm et al., 2017). A recent study has revealed that microglia can express CD11c under certain pathological conditions, which raises the debate over whether CD11c^+^ cells should be classified as a microglia subpopulation (Shen et al., 2022). While CD11c is not a specific marker for microglia, its expression can be upregulated in microglia under certain pathological or inflammatory conditions, making it a context-dependent indicator that should be interpreted in conjunction with more specific microglial markers (e.g., *Iba1*, *P2RY12*, or *TMEM119*).

In 1986, Hogg et al. discovered a subpopulation of microglia with dendrite-like morphology in the human brain, marking the first observation of CD11c on human microglia. The following research found a significant increase in the expression of CD11c in reactive microglia in the brains of AD patients. Subsequent immunohistochemical techniques confirmed that resting microglia also expressed CD11c (AkiyamaL and McGeer, 1990). These findings align with the leukocyte origin and phagocytic function of microglia while confirming the presence of inflammatory responses in AD.

The specific markers of microglia discovered in current research (such as TMEM119 and P2RY12) have already been helpful in distinguishing microglia from other myeloid cells under physiological and pathological conditions. Microglia have long been regarded as bone marrow-derived macrophages because they share markers such as CD11b, F4/80, CX3CR1 and IBA1(Gautier et al., 2012). The expression of CD11c in microglia has changed this perception. CD11c^+^ microglia subsets were identified as CD45^low^CD11b^+^CD11c^+^ cells in models such as experimental autoimmune encephalomyelitis (EAE) and optic neurodegeneration. They do not express the invasive leukocyte marker CCR2, but highly express CX3CR1, further confirming their microglial identity (Wlodarczyk et al., 2015, 2017). The relatively low expression of CD45 is a distinctive feature of microglia.

In 2006, Butovsky et al. finally proved that CD11c^+^ cells in CNS are microglia. They found that these cells expressed insulin-like growth factor 1 (IGF-1) under specific conditions. Their study revealed that glatiramer acetate vaccination can induce a phenotypic shift in microglia, transforming them into CD11c^+^ cells. This transformation led to a significant reduction in Aβ plaques and promoted neurogenesis, thereby enhancing the cognitive function of AD transgenic mouse models (Butovsky et al., 2006). Interleukin-4-like growth factor 1 interleukin-4-induced CD11c^+^ microglia could counteract the harmful effects of Aβ on adult neural stem/progenitor cells (Butovsky et al., 2006).

**Figure 1:**
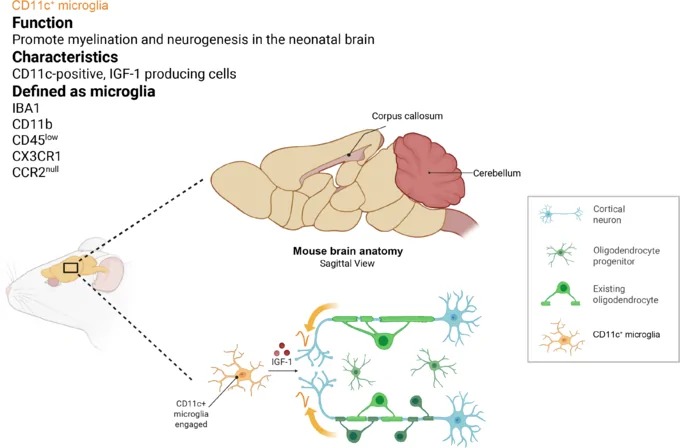
Characteristics of CD11c^+^ microglia. During development, CD11c^+^ microglia are specifically localized within the corpus callosum and cerebellum. Notably, they serve as a prominent source of IGF-1 and play a pivotal role in the processes of neurogenesis and myelination (Wlodarczyk et al., 2017). Created with BioRender.com. IGF-1: Insulin-like growth factor 1.

A recent study has discovered a transient population of CD11c^+^ microglia in the brains of newborn mice. This subset forms due to the phagocytosis of apoptotic neurons before birth and peaks between postnatal days 3 and 5 (P3 and P5) (Shen et al., 2022). It drops to an almost undetectable level in adults. These cells are predominantly located in regions of primary myelination, such as the corpus callosum and cerebellar white matter. Gene ontology analysis has linked CD11c^+^ microglia to neurogenesis and myelination, with IGF-1 being their major secreted product. Research has found that deficiency in IGF-1 can disrupt the myelin formation process at a critical stage of CNS development (Wlodarczyk et al., 2017). This phenomenon directly demonstrates the core regulatory role of CD11c^+^ microglia in neural development (**Figure 1**).

The number of CD11c^+^ microglia is dynamically changing throughout the life process. These cells appear during early development, decrease with age, and are almost non-existent in the CNS of healthy adults. Under aging or pathological conditions, their numbers increase significantly again (Benmamar-Badel et al., 2020). We suggest that these cells be collectively referred to as “CD11c^+^ microglia” given their unique markers and special functions.

## CD11c^+^ Microglia in the Healthy Central Nervous System

**Figure 2:**
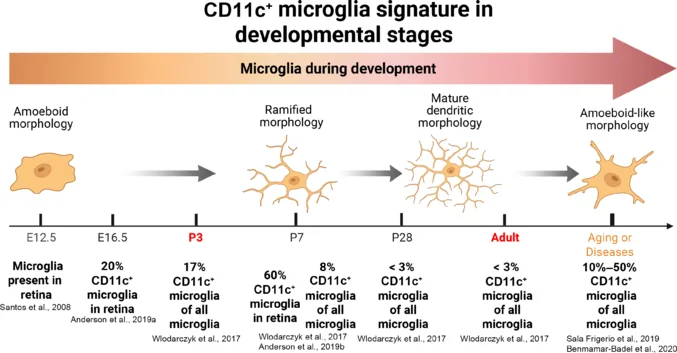
Characteristics of CD11c^+^ microglia across various developmental stages. As early as the 11.5-day embryo (E11.5) (Santos et al., 2008), microglia are identifiable in the mouse retina, branching out by E12.5 and exhibiting an amoeboid morphology. By E16.5, 20% of retinal microglia express CD11c (Anderson et al., 2019a). At postnatal day 3 (P3), CD11c^+^ microglia constitute 17% of all microglia in the mouse brain, decreasing to 8% by P7 (Wlodarczyk et al., 2017). However, in the retina, their proportion rises to 60% by this stage (Anderson et al., 2019b) and they display a more elaborate dendritic branching pattern. With advancing age, the percentage of CD11c^+^ microglia in the brain diminishes further, falling below 3% from P28 onwards and continuing this trend into adulthood, where they adopt a complex, highly branched dendritic appearance (Wlodarczyk et al., 2017). In old age or during disease states, the proportion of CD11c^+^ microglia expands to 10%–50%, accompanied by enlarged cell bodies, shorter and thicker branches, and reduced processes (Sala Frigerio et al., 2019; Benmamar-Badel et al., 2020). Created with BioRender.com. E: Embryonic; P: postnatal.

Although CD11c^+^ microglia are mainly observed under pathological conditions, their role in the development and homeostasis of CNS is also indispensable. Studies (Wlodarczyk et al., 2017; Nomaki et al., 2024) have shown that CD11c^+^ microglia exhibit regional and temporal variability during development. Their proportion peaks at P4 and P7, accounting for approximately 50% of the total microglia in the olfactory bulb and cerebellum. It decreased sharply after P14 and stabilized at a lower level in adulthood (Nomaki et al., 2024; **Figure 2**). To investigate the proportional dynamics of CD11c^+^ cells in the CNS, they utilized Itgax-Venus mice to effectively detect CD11c^+^ cells by expressing the fluorescent protein Venus, which is a reliable tool for studying CD11c^+^ microglia (Nomaki et al., 2024). These cells are mainly located in the white matter area, especially in the area where primary myelin formation occurs. Immunohistochemical analyses have revealed that all CD11c^+^ microglia co-express IBA1 and CX3CR1 and are situated within the brain parenchyma rather than in vascular or perivascular spaces (Wlodarczyk et al., 2017). They are the main sources of IGF-1, which is crucial for myelination. Moreover, they express genes associated with neurogenesis and myelination, including *Spp1*, *Lgals1*, and *Gpx3* (Wlodarczyk et al., 2017; Mehl et al., 2022).

In addition, studies have described the characteristics of microglia in the mouse retina during development and observed that 60% of the microglia in the P7 retina express CD11c (Anderson et al., 2019a, b; **Figure 2**). This might be because large developmental apoptotic waves occur in retinal neurons during the first week after birth, and exposure to apoptotic cells can trigger DAM-related microglial gene expression (Ayata et al., 2018). Apoptosis of retinal cells in the early postnatal period promotes the development of genetic traits related to aging and disease in microglia, independent of CSF1R signaling (Anderson et al., 2019a).

Microglia play an indispensable role in the development and maintenance of the brain throughout life (Prinz et al., 2021). As resident immune cells of the CNS, they not only act as immune sentries but also actively participate in the construction and maturation process of neural networks. The specific functions include regulating the number of neurons, guiding and refining synaptic formation, promoting myelin formation, regulating synaptic transmission efficiency and maintaining the excitability of neurons (Gallo et al., 2022; McNamara et al., 2023). In recent years, single-cell transcriptomic studies (Silvin et al., 2022; Lawrence et al., 2024) have revealed significant molecular and functional heterogeneity of microglia in different developmental stages (prenatal, postnatal, senescence and disease states) and brain regions (such as white matter and cortex). Under physiological conditions, the heterogeneity of microglia spans the prenatal and postnatal periods and has been named ATM (Hammond et al., 2019), PAM (Li and Barres, 2018), YAM (Silvin et al., 2022) or CD11c^+^ microglia (Wlodarczyk et al., 2017) in different studies. Their transcriptomic characteristics are the expressions of *Spp 1*, *Lgals3*, *Gpnmb*, *Clec7a*, *Itgax*, *Csf 1* and *Igf 1*, which are extremely similar to DAM, suggesting that there may be functional continuity between development and pathological processes (Silvin et al., 2022). Researchers explored the heterogeneity of microglia during the embryonic period by analyzing the single-cell transcriptome data of E9-E18 mouse embryos. Three functionally specialized subgroups were identified: circulating, non-circulating, and embryo-specific microglia (Lawrence et al., 2024). This study focuses on the developmental stage E14.5, at which point the spatial distribution of microglia in the forebrain shows significant heterogeneity (Thion and Garel, 2017). Akinde Rene et al. labeled all macrophages with Cx3cr1^gfp^ transgenic mice and found that microglia with an ATM-like phenotype were highly enriched at the fetal cortico-striato-amygdalar boundary (CSA) and cortico-septal boundary (CSB). These regions serve as transition zones between the neocortex and adjacent brain structures (Lawrence et al., 2024). The aggregation of ATM-like microglia occurs earlier than other glial cells and myelin formation processes. Macrophage and microglia exhaustion was induced by targeting the CSF1R signaling pathway (Pridans et al., 2018). The absence of microglia led to large “cavitary lesions” in CSA starting from E14.5 and persisting until E18.5 (Thion et al., 2019). These lesions specifically developed in regions where ATM-like microglia are typically present. Similar results were also obtained in CSB. Whole-brain iDISCO transparency and MRI imaging techniques further confirmed that these structural defects originated from the physiological absence of microglia rather than tissue processing artifacts. This result indicates that ATM-like microglia are crucial for maintaining the structural integrity of the CSA/CSB region (Lawrence et al., 2024). Notably, ATM-like microglia have been described as populations with similar phenotypes to CD11c^+^ microglia in different studies (Hammond et al., 2019; Silvin et al., 2022). The latter can ensure the stability of the fetal cortical boundary by inhibiting cavitary lesions induced by mechanical stress under physiological conditions.

A subsequent study has found that there are cell populations expressing CD11c in the brains and retinas of adult mice, which have a morphology similar to microglia but have been misjudged as DCs (Dando et al., 2016). This type of cells is mainly distributed in the ventral brain region, white matter tract, and hippocampal neurogenesis area. Single-cell mass spectrometry analysis confirmed the existence of CD11c^+^ microglia subsets in the human subventricular area and thalamus, which specifically express TMEM119 and P2RY12(Böttcher et al., 2019). This subset is virtually absent in healthy adult brains but increases dramatically under inflammatory conditions, potentially offering protective functions. Furthermore, CSF1R ligands such as CSF1 and IL-34 can drive the expansion of CD11c^+^ microglia (Immig et al., 2015; Boland and Kokiko-Cochran, 2024). These CD11c^+^ cells may originate from bone marrow–derived cells and local microglia, exhibiting characteristic low Class II histocompatibility complex (MHC-II) expression at homeostasis (Immig et al., 2015).

Aging can lead to changes in the quantity, state, gene expression, and phenotype of microglia. The number of microglia in the substantia nigrostriatal system and cortex of aged rats has decreased (Edler et al., 2021), while the hippocampal region remains unchanged (Edler et al., 2021). In contrast, the hippocampal region of rhesus monkeys shows increased density and enhanced phagocytic activity (Edler et al., 2021). Although the total number of cells remains stable, senescent microglia exhibit morphological degeneration, resulting in weakened neuroprotective functions (Silvin et al., 2023; La Sala and Farini, 2025). There are upregulated genes involved in host defense and neuroprotection during aging (Stanley et al., 2025), suggesting that changes in microglial phenotype may be linked to the expression of cell surface markers such as *Itgax*, *Lgals3*, *Axl*, *Clec7a*, *MHC-II*, and *Cxcr4* (Stanley et al., 2025). This series of phenotypic changes indicates that microglia undergo complex activation state transitions during aging and may be involved in immune surveillance and neural repair.

CD11c^+^ microglia have spatiotemporal specific distribution characteristics in the CNS. During the neonatal period, these cells promote myelin formation and neural development through the expression of specific genes. They almost disappear as the individual matures, but can re-expand and participate in tissue repair when encountering nerve damage or degenerative diseases. Aging causes changes in the number of microglia, and simultaneously triggers significant remodeling of their phenotype and transcriptome, leading to the CD11c^+^ subset exhibiting characteristics related to developmental abnormalities and neurodegeneration.

## CD11c^+^ Microglia in Neurological Diseases

**Figure 3:**
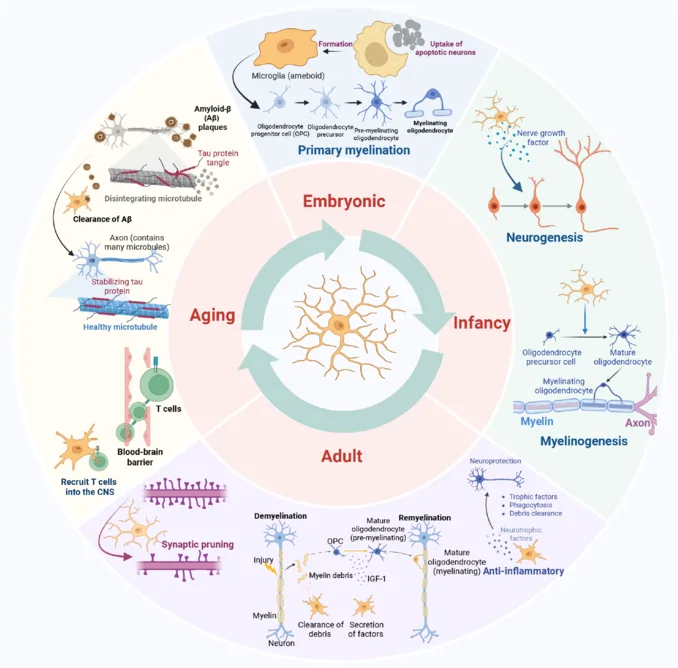
Role of CD11c^+^ microglia in different diseases. The CD11c^+^ microglial subpopulation originates prenatally following the uptake of apoptotic neurons (Shen et al., 2022) and is vital for primary myelination (Wlodarczyk et al., 2017). These cells are intricately linked to neonatal neurogenesis and myelination, serving as key players in neurodevelopment (Wlodarczyk et al., 2017). In adulthood and later life stages, particularly during disease states, CD11c^+^ microglia play a crucial role through their anti-neuroinflammatory effects (Böttcher et al., 2019), phagocytosis of myelin debris to facilitate myelin regeneration (Ponomarev et al., 2005), synaptic pruning (Shen et al., 2022), recruitment of T cells (Herz et al., 2015), and clearance of Aβ (Manczak et al., 2009; Reddy et al., 2009). Created with BioRender.com. Aβ: Amyloid-β; CNS: central nervous system; IGF-1: insulin-like growth factor 1; OPC: oligodendrocyte precursor cell.

The emergence of CD11c^+^ microglia subsets has been widely observed in the pathological processes of various neurological diseases, including neuroinflammation, demyelinating lesions, neurodegenerative diseases, and traumatic brain/spinal cord injuries (**Figure 3** and **Box 1**). Although the overactivation of microglia may trigger harmful reactions, this specific subpopulation demonstrates unique neuroprotective and tissue repair potential. This section will systematically analyze the core functions of CD11c^+^ microglia in these diseases and their potential therapeutic value.

Box 1: Role of CD11c^+^ microglia in different diseases

**(1) Neurodegenerative diseases**

**Research findings**

CD11c^+^ microglia demonstrate multifaceted neuroprotective functions across neurological disorders: they mediate amyloid-β clearance, regulate neuroinflammation, and improve metabolic environments in Alzheimer’s disease (Qiu et al., 2023; Lopes et al., 2024; Tsering et al., 2025); act as carriers of α-synuclein for brain–gut axis communication with heightened phagocytic activity in Parkinson’s disease (McFleder et al., 2023; Ma et al., 2025); and clear pathological proteins while phagocytosing myelin debris in amyotrophic lateral sclerosis (Xie et al., 2022).

**Practical significance**

These findings reveal the multiple roles of CD11c^+^ microglia in neurodegenerative pathology, including protein clearance and inflammation modulation, clarifying their mechanistic contributions to disease progression.

**(2) Stroke**

**Research findings**

In ischemic stroke, CD11c^+^ microglia directly facilitate white matter repair through remyelination promotion (Jia et al., 2023).

**Practical significance**

Research findings demonstrate the reparative function of CD11c^+^ microglia in white matter recovery post-stroke, highlighting their role in neural tissue restoration.

**(3) Central nervous system infectious diseases**

**Research findings**

For CNS infections, CD11c^+^ microglia orchestrate pathogen-specific immune responses; exert anti-inflammatory regulatory and phagocytic effects in viral encephalitis (Hincelin-Mery et al., 2023; Kumar et al., 2024); mediate the inflammatory response through CD11c/CD18 signaling in Nguyen virus disease (Carroll et al., 2023); regulate the interaction between parasites and effector T cells in the immune response during Toxoplasma encephalitis (Suzuki et al., 2005); mediate immune responses through Toll-like receptors 1/2 signaling in Lyme disease (Cassiani-Ingoni et al., 2006); and participate in the immune response through the formation of microglial nodules in long-term COVID-19 sequelae (Schwabenland et al., 2024).

**Practical significance**

These findings confirm their indispensable role in immune coordination and pathogen clearance during central nervous system infections.

**(4) Demyelinating diseases**

**Research findings**

In demyelinating pathologies, CD11c^+^ microglia simultaneously modulate immunity and drive regeneration: mediating immune regulation and remyelination in multiple sclerosis (Ponomarev et al., 2005), and reducing inflammation while promoting tissue repair in experimental autoimmune encephalomyelitis (Kim et al., 2018).

**Practical significance**

This clarifies their dual function as both immune modulators and regenerative agents in demyelinating pathologies.

**(5) Neuropathic pain**

**Research findings**

In neuropathic pain, CD11c^+^ microglia phagocytose myelin debris and express insulin-like growth factor 1, directly linking to myelin-associated pain mechanisms (Kohno et al., 2022).

**Practical significance**

Research findings directly link CD11c^+^ microglia to myelin-associated pain mechanisms via insulin-like growth factor 1-dependent pathways.

**(6) Traumatic brain injury or spinal cord injury**

**Research findings**

In traumatic neural injury, CD11c^+^ microglia suppress secondary damage by regulating complement signaling in traumatic brain injury (Iannucci et al., 2022) and dampening neuroinflammatory cascades in spinal cord injury (Donnelly et al., 2011).

**Practical significance**

Research findings identify them as key modulators of inflammatory and signaling cascades in secondary injury responses.

### CD11c^+^ microglia in neurodegenerative diseases

CD11c^+^ microglia are intimately associated with the pathological processes of several neurodegenerative diseases, notably AD, PD, and amyotrophic lateral sclerosis (ALS). These cells frequently accumulate around key pathological sites, such as amyloid plaques or α-synuclein aggregates, where they exhibit distinct transcriptional profiles and functional roles.

#### CD11c^+^ microglia in Alzheimer’s disease

Early evidence indicated that reactive microglia in the brains of AD patients display significantly elevated CD11c expression (Akiyama and McGeer, 1990). Combined analysis by immunohistochemistry and electron microscopy revealed that in the brain tissues of AD patients, CD11c^+^ perivascular cells (possibly microglia or macrophage-like cells) were mainly aggregated in the Aβ deposition area. Although there is no significant difference in the overall distribution density between normal and pathological brain tissues, specific spatial localization suggests that these cells may be involved in the pathological mechanisms of AD, including Aβ clearance or local microenvironment regulation (Sasaki et al., 1996).

From the perspective of spatial distribution, CD11c^+^ microglia exhibit a unique aggregation pattern in the middle and late stages of AD. They not only focus on plaque distribution, but also form dynamic gene expression profiles by upregulating genes related to immune regulation, lysosomal function and metabolism (Tsering et al., 2025). This heterogeneity enables CD11c^+^ microglia to perform differentiated functions at different stages of the disease. The early neuroprotective effect of CD11c^+^ microglia is exerted through phagocytosis of Aβ and inhibition of inflammation (Kamphuis et al., 2016). However, some cells may transform into pro-inflammatory phenotypes, thereby exacerbating nerve damage during the progression of the disease (Ramesha et al., 2021). Lopes et al. (2024) investigated the role of *Itgax* and *Spp1* in the cerebral cortex of the older adult individuals with AD and related diseases. At single‐cell resolution, Itgax was highly expressed in microglia, where specific subpopulations were associated with AD and cerebral amyloid angiopathy (Lopes et al., 2024).

The activity of CD11c^+^ microglia is regulated by multiple signaling pathways in terms of functional mechanisms. The TLR2 signaling pathway mediates its immune regulation of Aβ and drives inflammatory responses (Jana et al., 2008). The granulocyte-macrophage colony-stimulating factor signaling pathway affects cell migration and Aβ clearance efficiency (Manczak et al., 2009; Reddy et al., 2009). In addition to regulating the activation status of CD11c^+^ microglia, these signaling pathways also affect their functional performance and behavioral characteristics under pathological conditions.

CD11c^+^ microglia mediate the transformation of inflammation to the neuroprotective state by secreting IGF-1, thereby promoting neurogenesis and tissue repair (Butovsky et al., 2006). The study by Butovsky et al. (2006) suggested that such cells mainly originated from bone marrow. However, Wu et al. (2021) indicated that CD11c^+^ microglia retain embryonic lineage characteristics in the pathological microenvironment of AD. They maintain the lineage characteristics of embryonic origin and rely on the self-renewal of local microglia to maintain their existence in the late stage of the disease. This phenomenon highlights the stability of microglia in the pathological process of AD and their ability to regulate dynamic functions.

The role of CD11c^+^ microglia in AD is dual, and their functional heterogeneity hinders a comprehensive analysis of their pathological contributions. Qiu et al. (2023) found that the expression of osteopontin (OPN) can distinguish CD11c^+^ microglia into pathogenic (CD11c^+^OPN^+^) and protective (CD11c^+^OPN^−^) subpopulations (Qiu et al., 2023). The generation of OPN activates the pro-inflammatory phenotype and may inhibit the clearance and integration of Aβ by interfering with the TREM2/TAM-lysosomal phagocytic pathway. Experiments have shown that targeted inhibition of OPN can reduce the number of pro-inflammatory microglia, alleviate Aβ deposition, and improve cognitive function (Qiu et al., 2023; Oshima et al., 2024). Analysis of brain tissue from AD patients indicates that levels of OPN-producing CD11c^+^ microglia correlate strongly with the degree of cognitive deficit and AD neuropathology (Qiu et al., 2023). It is suggested that OPN may be a potential therapeutic target for AD immune intervention.

Overall, CD11c^+^ microglia exhibit highly dynamic biological characteristics in the pathogenesis of AD. On one hand, CD11c^+^ microglia exert neuroprotective effects by enhancing Aβ phagocytosis, inhibiting excessive inflammation, and optimizing the metabolic environment. On the other hand, phenotypic transformation may cause it to change into a pro-inflammatory subtype and exacerbating nerve damage. This functional duality highly depends on the disease stage and the characteristics of the microenvironment.

#### CD11c^+^ microglia in Parkinson’s disease

The main pathology of Parkinson’s disease (PD) is the self-assembly of alpha-synuclein (αSyn) monomers to form various toxic aggregates, including oligomers, protofibrils, and fibrils (Chen et al., 2025c; Cui et al., 2025). Like in AD, microglia activation and the associated neuroinflammatory response are not merely bystanders but active contributors to PD pathogenesis. CD11c^+^ microglia also play a pivotal regulatory role in PD. McFleder et al. (2023) have shown that this type of cells is closely related to neuroinflammatory responses and abnormal aggregation of αSyn. In mouse models expressing human αSyn mutants, CD11c^+^ microglia mediate the bidirectional transport of αSyn between the CNS and the gut, establishing a bidirectional regulatory mechanism of the brain–gut axis (McFleder et al., 2023). Single-cell RNA sequencing further revealed that there are CD11c^+^ cell subpopulations with similar phenotypic characteristics in the brain and intestine, which showed a significant activation state in the PD model (McFleder et al., 2023). These findings not only clarify the core role of CD11c^+^ microglia in the pathological spread of αSyn and neuroinflammation, but also provide a new direction for the mechanism research and targeted therapy of PD. This study reveals a novel pathological transmission mechanism of PD: CD11c^+^ cells activated by αSyn in the brain can act as a “Trojan horse”, carrying and transporting pathological αSyn protein from the brain to the intestine, thereby sowing pathology in distant organs and causing corresponding functional disorders. This “brain–gut axis” communication mediated by CD11c^+^ cells provides an important theoretical basis for understanding the systemic progression of PD and developing new therapeutic targets, such as preventing the migration or function of these cells.

#### CD11c^+^ microglia in amyotrophic lateral sclerosis

ALS, also known as Lou Gehrig’s disease, is a chronic and relentlessly progressive neurodegenerative disorder primarily targeting motor neurons (Larrea et al., 2025). Accumulating evidence suggests that the abnormal activation of microglia plays a pivotal role in ALS pathology.

In the SOD1G93A mouse model (carrying human Cu/Zn-superoxide dismutase mutations), Krasemann et al. (2017) observed that disease progression was accompanied by a significant increase in the expression level of *Itgax*. This phenomenon marks the transformation of microglia from the M0 phenotype that maintains homeostasis to the MGnD phenotype related to neurodegeneration. This transition is characterized by the downregulation of homeostatic genes (e.g., *P2ry12*, *Tmem119*) and the upregulation of inflammation-associated genes (e.g., *Itgax*, *Apoe*). The sustained high expression of *Itgax* on the surface of microglia reflects its inflammatory activation status in the pathology of ALS (Krasemann et al., 2017).

Furthermore, Xie et al. (2022) found a significant increase in the number of CD11c^+^ microglia subsets in the ALS model expressing human TPP-43 protein. CD11c^+^ microglia exhibit enhanced phagocytic activity and can effectively clear pathological TDP-43 aggregates. The study by Xie et al. (2022) found that the emergence of CD11c^+^ microglia is closely related to the *TREM2* signaling pathway. In the mouse model with *TREM2* gene deletion, the number of this subpopulation was significantly reduced (Xie et al., 2022). CD11c^+^ microglia were almost undetectable under normal physiological conditions. However, it significantly amplified in the pathological environment of ALS, and its quantity was positively correlated with the severity of the disease (Xie et al., 2022). These findings suggest that CD11c^+^ microglia are a dynamic subpopulation driven by the disease microenvironment and play a key role in the regulation of neuroinflammation and the clearance of pathological proteins.

### CD11c^+^ microglia in ischemic stroke

In contrast to the progressive nature of AD, PD, and ALS, ischemic stroke (IS) causes rapid brain damage, yet similarly involves the robust activation of CD11c^+^ microglia in the infarct and peri-infarct regions. White matter injury and neurological dysfunction caused by IS have been widely verified (Jia et al., 2023). CD11c^+^ cells persist in the area around the lesion for several weeks in IS. It is mainly distributed in non-core lesion areas such as degenerated corticothalamic tracts and subcortical nuclei. All CD11c^+^ cells presented a surface marker of CD11b^+^/CD8α^–^/CD205, indicating their myeloid origin characteristics. It is worth noting that a small portion of these cells exhibit a CD45^low^ phenotype, which is consistent with the labeling characteristics of *in situ* microglia (Jia et al., 2023).

The CD11c^+^ cell population in IS is heterogeneous, comprising both CNS-resident microglia and peripherally derived myeloid cells. In the mouse model of transient middle cerebral artery occlusion (tMCAO), white matter injury peaked on the 7^th^ day after stroke and gradually recovered from the 7^th^ to the 30^th^ day. Studies have shown that microglia (especially the CD11c^+^ subset) play a significant role in the repair process (Cao et al., 2021; Jia et al., 2023). These cells mainly gather in the damaged myelin sheath area and promote the formation of new myelin sheaths by phagocytosing myelin fragments. The number of CD11c^+^ microglia continued to increase from the 7^th^ day to the 30^th^ day, and their phagocytic activity was significantly enhanced. It simultaneously shows the upregulation of phagocycle-related genes (such as *Csf1r* and *Cd68*) and lipid metabolism genes (such as *Lpl* and *Abca1*) (Jia et al., 2023). These molecular changes may promote the recovery of neurological function by supporting oligodendrocyte maturation and myelin regeneration. The above results indicate that CD11c^+^ microglia have dual functions in white matter repair. On one hand, pathological fragments are removed to alleviate inflammatory responses; on the other hand, myelin sheath reconstruction and systemic neural recovery are supported through gene regulatory networks.

Ju et al. (2022) found that monocyte-derived macrophages (MDMs) underwent dynamic phenotypic transformation after infiltrating ischemic brain tissue. CD45^High^ MDMs gradually evolved to the CD45^Low^ microglia-like phenotype by phagocytosing apoptotic cells and metabolic debris (Ju et al., 2022). In the ischemic injury area, CD11c^+^ cells are mainly distributed in the CD45^High^ MDM subpopulation. This type of CD11c^+^ MDMs exhibits significant proliferative advantages and enhanced phagocytic ability, and can promote tissue repair by efficiently eliminating dead cells and pathological debris. Moreover, phagocytic activity is positively correlated with the conversion ratio from CD45^High^ to CD45^Low^, suggesting that phagocytosis may drive this phenotypic transformation process (Ju et al., 2022).

Astrocyte-secreted Flt3 ligand has been shown to promote the development and expansion of CD11c^+^ cells following cerebral ischemia, which include populations derived from microglial proliferation and infiltrating DCs (Gallizioli et al., 2020). While these cells share some markers and morphological characteristics, they exhibit significant differences in the expression of pattern recognition receptors and chemokine receptors. The pronounced aggregation of CD11c^+^ microglia and MDMs in the degenerative thalamus following acute ischemia suggests a critical role in post-stroke pathological changes and neurological repair (Cao et al., 2021). Despite their distinct origins, both resident CD11c^+^ microglia and infiltrating CD11c^+^ MDMs converge toward a phagocytic, repair-promoting phenotype, highlighting their complementary roles in post-stroke recovery.

Overall, CD11c^+^ microglia actively participate in brain injury responses and support subsequent tissue repair through phenotypic transformation, proliferation, phagocytosis, and immune regulation.

### CD11c^+^ microglia in central nervous system infectious diseases

The robust activation of CD11c^+^ microglia observed in sterile injuries such as stroke also occurs in response to pathogen-associated molecular patterns during CNS infections. Infections of CNS encompass acute or chronic inflammatory (or non-inflammatory) diseases caused by various pathogens, which carry a high disease burden (Dias et al., 2025). Common pathogens include viruses, bacteria, fungi, spirochetes, mycoplasma, parasites, rickettsiae, and prions (Venkatesan and Geocadin, 2014). Microglia are regarded as the first line of defense against brain infections. CD11c^+^ microglia participate in multiple stages of the antiviral response, including pathogen sensing, phenotypic activation, and crosstalk with adaptive immunity—processes illustrated in the following examples.

A variety of viruses can infect CNS of mammals and cause neurological dysfunction. Due to the limited tolerance of CNS to immune responses, eliminating persistent neurotropic viral infections has become a key challenge. In the model of persistent infection with lymphocytic choroidal meningitis virus, Knudson et al. (2021) found that therapeutic antiviral T cells can not only effectively clear the virus, but also maintain the integrity of the BBB and avoid tissue damage. This process relies on the targeted recruitment of chemokine-driven specific T cells, while inhibiting the infiltration of pathogenic innate immune cells such as neutrophils. Notably, the interaction between these T cells and CD11c^+^ microglia activates the STAT1 signaling pathway, enabling non-cytolytic viral clearance (Herz et al., 2015), highlighting a key role for microglia in coordinating adaptive immunity.

Chikungunya virus (CHIKV), as a key pathogen causing CNS complications, its infection may lead to encephalitis and related nerve damage. Kumar et al. (2024) found that CHIKV has a significant phenotypic regulatory effect on human microglia (C20 cell line). Experimental observations revealed that C20 cells after infection presented pathological features such as mitochondrial dysfunction and increased apoptosis, accompanied by specific changes in surface molecule expression. The expression levels of CD11c and HLA-DR were significantly increased, while the expression of CD14 was significantly decreased. Specifically, the upregulation ratio of CD11c in infected cells reached 67.7%, the proportion of HLA-DR positive cells was 8.5%, and only 4% of the cells still maintained CD14 expression (Kumar et al., 2024). These findings suggest that CHIKV may modulate CNS inflammatory responses and injury repair by reshaping microglial phenotype, particularly through sustained CD11c activation.

Prion diseases result from abnormal folding of host proteins, leading to glial cell proliferation, inflammatory responses, and neurodegenerative changes. Carroll et al. (2023) discovered that microglia respond to prion invasion through CD11c/CD18 (an integrin-receptor complex) mediated signaling pathway. However, mouse models with CD11c deletion showed no significant differences in prion-related pathological features (such as glioma formation and viral protein aggregation) compared with normal mice, suggesting that CD11c may not be a necessary condition for microglia to exert neuroprotective functions (Carroll et al., 2023). RNA sequencing analysis revealed that in the terminal stage of prion disease, microglia form a unique molecular expression profile, suggesting that CD11c signaling may be involved in regulating the inflammatory process at a specific stage (Carroll et al., 2020).

West Nile virus infection is another important cause of viral encephalitis. Thammahakin et al. (2023) have shown that west Nile virus infection can induce the activation of microglia, manifested as cell morphological remodeling and the emergence of DAM. At the infection site, CD11c^+^ microglia showed a significant increase in the low expression level of *Tmem119* (Thammahakin et al., 2023). This DAM phenotype may contribute to disease progression through inflammatory and phagocytic activities, though the precise mechanisms require further investigation.

During Toxoplasmosis encephalitis (TE), CD11c^+^ cells in the CNS mainly include microglia and DCs. These cells migrate to the CNS through multiple chemokine receptor pathways and remain long-term during the infection process (John et al., 2011). CD11c^+^ microglia can come into direct contact with parasites and effector T cells and participate in immune responses (Suzuki et al., 2005). Together, they contribute to immune regulation and tissue repair following parasitic infection.

In the infection model of neurotropic mouse hepatitis virus (MHV-JHM strain), CD11c^+^ microglia gradually emerged as the disease progressed. Viral infection promotes the transformation of monocytes into macrophages and induces the upregulation of CD11c expression (Templeton et al., 2008). This type of cells exhibits the characteristics of mature antigen-presenting cells and is closely related to myelin damage and axon injury, suggesting that they may be involved in the pathogenic process by regulating inflammatory responses or exacerbating myelin damage (Templeton et al., 2008). These CD11c^+^ cells mainly originate from peripheral monocyte infiltration and differentiate into CD11c-expressing macrophages or microglia in the CNS (Templeton et al., 2008). They are key players in inflammation regulation and may act as direct effectors of myelin injury.

Lyme disease is caused by Borrelia burgdorferi. About 20% of infected individuals will present with neurological symptoms (Cassiani-Ingoni et al., 2006). Microglia express CD11c for immune response under the stimulation of Borspira burgdorferi, which may depend on the activation of the TLR1/2 signaling pathway (Cassiani-Ingoni et al., 2006).

Acute COVID-19 and post-COVID-19 syndrome (PCC) can also have some obvious neurological symptoms (Davis et al., 2021). Schwabenland et al. (2024) found that persistent activation of microglia characterizes the CNS of post-COVID-19 patients, revealed a high percentage of TMEM119^+^P2RY12^+^CD68^+^Iba1^+^HLA-DR^+^CD11c^+^SCAMP2^+^ microglia assembled in prototypical cellular nodules. This phenomenon suggests that CD11c^+^ microglia may be involved in the neuroimmunity of PCC. Zhang et al. (2023b) pointed out that the immune response pattern of patients after COVID-19 may shift from adaptive immunity to innate immunity, and this transformation may become a potential inducement for CNS damage and long-term sequelae.

Microglia are the major reservoir of HIV-1 (HIV) within the CNS (Rawat and Spector, 2017). Rawat and Spector (2017) established a monocyte-derived microglia (MMG) cell model for HIV infection. They found that the infected MMG exhibited the characteristic of positive CD11c, and the persistently infected MMG can be used as an *in vitro* model for studying strategies to eliminate the virus from the CNS.

Based on the above research, CD11c^+^ microglia play multiple roles in CNS infectious diseases. These cells play a key role in tissue repair mechanisms by dynamically regulating immune responses and inflammatory processes.

### CD11c^+^ microglia in demyelinating diseases

The critical role of CD11c^+^ microglia in myelin development and maintenance has been widely verified. In the healthy neonatal brain, CD11c^+^ microglia are mainly distributed in the primary myelin formation area, showing specific neurogenic characteristics. Wlodarczyk et al. (2017) discovered that these cells play a supporting role in myelin structure formation by expressing key genes that regulate neuronal survival, glial cell migration and differentiation. The absence or functional abnormality of CD11c^+^ microglia may exacerbate myelin injury and impair the repair process.

In demyelinating diseases such as multiple sclerosis (MS), the destruction and insufficient regenerative capacity of myelin sheath pose a therapeutic challenge. Teo et al. (2023) found that the loss of function of ceramide synthase 2 (CerS2) would lead to a reduction of long-chain sphingolipids in myelin sheath, resulting in a decrease in the structural stability of myelin sheath and thereby exposing damaged myelin protein epitope. This abnormality can trigger the activation of CD11c^+^ microglia, prompting them to initiate a phagocytic response to clear the damaged myelin sheath. Similar phenomena are also observed in the corpus callosum demyelination model induced by copper. Microglia proliferate significantly in the lesion area and enter an activated state, manifested as enhanced expression of MHC molecules and CD11c. This characteristic change is similar to the function of antigen-presenting cells (Lee et al., 2022). CD11c^+^ microglia not only clear myelin fragments through phagocytosis, but also support oligodendrocyte maturation and myelin regeneration by releasing signal molecules that promote regeneration (Kim et al., 2018).

In models of MS and EAE, CD11c^+^ microglia have been confirmed to be important drivers of inflammatory responses and myelin sheath destruction. Mayrhofer et al. (2021) found that the reduction in the number of CD11c^+^ microglia was positively correlated with the clinical deterioration of EAE, while its functional activation was closely related to myelin regeneration and immune regulation.

It has been found that protein arginine methyltransferase 1 (PRMT1) is a core factor in regulating the gene cluster related to myelin regeneration of CD11c^+^ microglia, and its loss of function significantly affects the activation state of microglia. PRMT1-deficient microglia have difficulty forming activated subpopulations with high expression of MHC and CD11c. This phenotypic abnormality directly leads to decreased myelin regeneration ability and aggravates demyelinating lesions (Lee et al., 2022). Furthermore, the absence of the inflammatory regulatory factor miR-146a weakens the anti-inflammatory ability of microglia, manifested as a reduction in the number of CD11c^+^ cells, and thereby deteriorates the pathological features of demyelination (Martin et al., 2020). CD11c^+^ microglia were found to be activated in experimental neuromyelitis optica (NMO) through IFN-I signaling pathway. Their numbers increased significantly in the lesion area, indicating that they are involved in the regulatory process of NMO-specific inflammatory responses (Wlodarczyk et al., 2021).

In conclusion, CD11c^+^ microglia are the core immunomodulatory cells in demyelinating diseases. They actively participate in the inflammatory response during demyelination by eliminating damaged tissues and presenting antigens. It also plays a protective role in myelin regeneration by promoting the maturation of oligodendrocytes.

### CD11c^+^ microglia in neuropathic pain

In addition to being involved in neurodegenerative diseases and infections, CD11c^+^microglia are also closely related to the pathogenesis of neuropathic pain and play a key role in the transmission of nociceptive signals in the spinal cord. Neuropathic pain is a chronic disease caused by peripheral nerve injury, characterized by the persistence of pain symptoms even after the original injury has healed (Hiraga et al., 2022). The immune system plays a key role in the occurrence and development of neuropathic pain. Among them, spinal microglia significantly intensify pain perception by sensing neuronal activity and establishing positive feedback loops. Based on this mechanism, blocking the signal transmission between microglia and neurons has become a potential direction for analgesic intervention. Kohno et al. (2022) discovered a group of special CD11c^+^ microglia in the dorsal horn of the spinal cord, which appear after episodes of neuropathic pain and help relieve the pain. These microglia promoted pain recovery through their high expression of IGF-1 and phagocytosing myelin debris. The CD11c^+^ microglia remained even after pain recovery, and pain hypersensitivity returned if they were depleted (Kohno et al., 2022).

Donovan et al. (2024) further verified the role of microglia in pain by using the clinical model of tibial fracture and complex regional pain syndrome. They discovered that microglia depletion and repopulation at the acute-to-chronic transition completely resolved pain and reduced peripheral inflammation. Repopulated spinal cord microglia present specific morphological and transcriptional characteristics, with significantly upregulated *Itgax* expression (Donovan et al., 2024).

These findings clarify the mechanisms underlying the relief and recurrence of neuropathic pain and may contribute to the development of therapeutic strategies by regulating CD11c^+^ microglia.

### CD11c^+^ microglia in traumatic brain and spinal cord injury

The integrity of the CNS is violently breached in traumatic injuries. Traumatic brain injury (TBI) and spinal cord injury (SCI) are two common types of trauma in the CNS. Although there are anatomical differences in the injury sites, the two exhibit highly overlapping features during the pathological evolution process. The injury mechanism not only involves the initial physical tissue destruction (primary injury), but also the subsequent complex pathological processes, including secondary injuries such as inflammatory response, immune cell activation, oxidative stress and cell death (Toutonji et al., 2023; Al Mamun et al., 2024). CD11c^+^ microglia are also a key focus of research in this kind of trauma, and they are closely related to the functional changes of various immune cell types and injury mechanisms.

Research has found that the absence of signal-regulating protein α (SIRPα) and CD47 ligand both inhibit the proliferation of CD11c^+^ microglia in the white matter of the brain after TBI. It indicates that the interaction between SIRPα in microglia and CD47 on adjacent cells inhibits the formation of CD11c^+^ microglia (Sato-Hashimoto et al., 2019). The interaction between SIRPα on the surface of microglia and CD47 of adjacent cells can inhibit the generation of the CD11c^+^ phenotype, and such cells may have neuroprotective properties during the demyelination repair stage (Sato-Hashimoto et al., 2019).

Neuroinflammatory responses can persist for several years after TBI and lead to the development of chronic neurological manifestations. Complement plays a core role in neuroinflammation after TBI. The expression of TBI-modulated phagocytes and complement receptors in peripheral immune cells residing and infiltrating the brain, and the same cell populations within different functional clusters are identified at different stages after TBI. In particular, the CD11c^+^ (CR4) microglia subset continued to expand within 28 days after injury and was the only receptor that showed a sustained increase over time (Toutonji et al., 2023).

Zhang et al. (2020) found that microglia could transform into the CD11c^+^ phenotype in a cerebral ischemia-reperfusion injury model. This type of cells showed significant upregulation of co-stimulatory molecules such as MHC-II, CD80 and CD86. It is activated through IFN-γ and its downstream ERK/c-myc signaling pathway. This transformation augments the antigen-presenting ability of microglia and their capacity to activate naive T cells, thereby playing a fundamental role in neuroinflammation.

In diabetics models, persistent hyperglycemia and hypoxic environments can indirectly activate microglia (including CD11c^+^ subsets) by damaging brain microvascular endothelial cells, manifested as increased secretion of pro-inflammatory factors (such as TNF-α and MMP-9) and enhanced phagocytic activity. This pathological change may accelerate the progression of brain injury (Iannucci et al., 2022). In the rat model of cerebral palsy (CP), repeated infections can induce continuous activation of microglia, manifested as IBA1^+^ cell proliferation and dynamic changes of CD11c^+^ phenotype (Liu et al., 2022). This abnormal activation may have a significant impact on the formation and repair process of cerebral palsy.

Ellman et al. (2016) found that after SCI, tumor necrosis factor (TNF) derived from microglia in the damaged spinal cord exists in the form of membrane-bound TNF (mTNF) and cleaved soluble TNF (solTNF). Genetic ablation of solTNF, but sustained expression of mTNF significantly increased the number and expression levels of MHCII^+^ and CD11c^+^ microglia in the lesion area. It indicates that mTNF promotes an anti-inflammatory environment in the injured spinal cord, and it is speculated that this may be related to CD11c^+^ microglia (Ellman et al., 2016). After SCI, resident and peripherally derived CD11c^+^ monocytes have spatiotemporal specific recruitment characteristics at the injury site. Observations by *in vivo* two-photon microscopy revealed that these cells exhibited unique movement trajectories and phagocytic abilities (Fenrich et al., 2013). It is notable that defects in the CX3CR1 signaling pathway can significantly alter the proportion of CD11c^+^ cells and interfere with the neuroinflammatory process, suggesting its core position in spinal cord repair (Donnelly et al., 2011).

Collectively, CD11c^+^ microglia play multiple roles in TBI, SCI, and related diseases. Its functional dynamic changes are regulated by multiple factors such as the complement system, signal regulatory proteins, chemokines, and the local microenvironment.

## Potential of CD11c^+^ Microglia as a Therapeutic Target

Considering the beneficial evidence observed in the aforementioned studies (Jia et al., 2023; Qiu et al., 2023), CD11c^+^ microglia show great potential in the treatment of neurological diseases. CD11c^+^ microglia exhibit plasticity characteristics, allowing them to change their functional state according to different pathological environments. They are closely related to AD, PD, stroke, ALS, and demyelinating diseases. This opens up new prospects for targeted therapies involving microglia in neurological diseases.

### Omics-driven target discovery

#### Molecular characterization revealed by single-cell transcriptomics

With the advancement of technology, transcriptomics has evolved from low-throughput microarrays to high-throughput NGS and has ultimately reached single-cell resolution. Researchers have gradually achieved in-depth exploration from the population level to the single-cell level (Samelak-Czajka et al., 2024). Single-cell RNA sequencing (scRNA-seq) overcomes the limitations of traditional methods with its high sensitivity and comprehensive genome-wide transcriptome coverage (Qu et al., 2024). For the first time, it reveals the molecular characteristics of different cell subsets in complex tissues, providing a new perspective for analyzing cellular heterogeneity.

In the field of neuroscience, single-cell RNA sequencing (scRNA-seq) systematically elucidates the heterogeneity of microglia during development, homeostasis, and disease processes, particularly in combination with multi-omics techniques. Researchers have successfully identified characteristic marker genes of CD11c^+^ microglia subsets, such as Spp1, Igf1, and Clec7a (Depp et al., 2025; Sankowski and Prinz, 2025). These findings clarify the core role of this subgroup in maintaining fetal brain structure, synaptic pruning, and disease-related phenotypes (such as disease-associated microglia in AD), providing a molecular basis for understanding its functional regulatory network and pathogenic mechanisms (Martins-Ferreira et al., 2021, 2025). In terms of therapeutic target development, scRNA-seq is reshaping our understanding of CD11c^+^ microglia. In diseases such as AD and MS, the abnormal activation of these cells is closely related to disease progression. scRNA-seq can identify key effector genes within these cells, providing a new approach for precise intervention (Qiu et al., 2023). In the future, microglia-specific regulatory models can be constructed using gene editing technologies such as clustered regularly interspaced short palindromic repeats (CRISPR)/CRISPR-associated protein 9 to systematically analyze their functional characteristics under neuropathological conditions. Combining animal models with drug intervention experiments will provide a foundation for transforming therapeutic strategies targeting CD11c^+^ microglia.

#### Discoveries in proteomics and metabolomics

The unique protein expression profile of CD11c^+^ microglia provides a basis for understanding their functions and roles in neurological diseases. Proteomic analysis indicates that CD11c^+^ microglia demonstrated enhanced inflammatory responses and metabolic activities in AD models. It is specifically manifested as the upregulation of immune response and cell metabolism-related proteins (such as Neprilysin and lysosome-associated membrane protein 2A) (Gholampour et al., 2025). Furthermore, Barreto-Núñez et al. (2024) also revealed their adaptive changes in the microenvironment. These changes may be associated with nerve damage and disease progression.

Proteomics using high-throughput mass spectrometry technology can deeply analyze key biomarkers and potential therapeutic targets in CD11c^+^ microglia. Specific proteins such as S100A9 and Ppm1g, which are closely related to neuroinflammatory regulation, may bring new intervention directions for neurodegenerative diseases such as AD and ALS (Martin et al., 2020; Barreto-Núñez et al., 2024).

From a metabolic perspective, CD11c^+^ microglia undergo significant metabolic reprogramming under inflammatory conditions to meet their increased energy and synthetic demands. Multiple studies have observed enhanced glycolytic pathway activity and significant alterations in lipid metabolism pathways in AD (Altendorfer et al., 2025; Zhang et al., 2025b). These metabolic characteristics are closely related to the intensity of cellular inflammatory responses. Under stress conditions such as hypoxia or chronic inflammation, the levels of key metabolites such as lactic acid, amino acids, and fatty acids fluctuate significantly, reflecting the adaptive regulatory mechanism of microglia to complex microenvironments (Altendorfer et al., 2025; Zhang et al., 2025b). This change in metabolic regulation not only alters the way microglia obtain energy but also profoundly influences their immune function performance. The adjustment of metabolic pathways enhances the release and migration capabilities of cytokines when they transition from the resting state to the activated state. Neprilysin, as a significantly upregulated protein in AD models, may serve as a biomarker for evaluating disease progression (Walker et al., 2022; Gholampour et al., 2025).

Metabolomics studies have revealed the unique metabolic characteristics of microglia, and changes in their metabolite profiles can reflect the functional state of these cells (Benarroch, 2022; Jung et al., 2025). Microglia exhibit significant metabolic adaptability and can dynamically switch between oxidative phosphorylation and glycolysis (Jung et al., 2025). This fluctuation in metabolic levels can serve as an indicator to evaluate the activation status of microglia, determine the degree of neuroinflammation, and assess therapeutic effects (Jung et al., 2025). Glucose metabolism is essential for maintaining the function of microglia (Sadeghdoust et al., 2024). Under homeostatic conditions, microglia primarily rely on mitochondrial oxidative phosphorylation for energy supply. However, they rapidly shift to the glycolytic pathway in response to inflammation, a metabolic pattern conversion that meets the energy demands of a swift immune response (Cheng et al., 2021).

Lipids are important immunomodulatory molecules that play a key role in maintaining cellular health and function. Microglia regulate the extracellular environment and intracellular lipid metabolism through various mechanisms. Julia et al. found that the abnormal accumulation of lipid droplets (LD) caused by Aβ deposition impairs phagocytic function and increases the generation of reactive oxygen species (ROS), thereby promoting neuroinflammation in Alzheimer’s disease (Prakash et al., 2025). Risk genes such as *Trem2* and *Apoe* in microglia are considered to be deeply involved in lipid metabolism (Li et al., 2022).

In addition to glucose and lipids, microglia can also replenish energy through amino acid metabolism, especially demonstrating metabolic flexibility when glucose supply is limited (Kelly and Pearce, 2020). However, excessive activation of microglia occurs when amino acid metabolism is imbalanced, especially in individuals carrying the APOE4 allele (Hollinger et al., 2020). This disorder can significantly aggravate the neuroinflammatory response. The discovery of these metabolic characteristics provides new markers for the early screening of neurological diseases and can help formulate personalized treatment plans.

### Neural regeneration–oriented intervention strategies

#### Regulatory methods for promoting myelin regeneration

It is known that the pro-remyelination properties of microglia in the CNS. CD11c^+^ microglia exist in the corpus callosum of myelin regeneration and express several characteristic genes (*Itgax*, *Igf1*, *Clec7a*, *Apoe*, and *Spp1*) (Benmamar-Badel et al., 2020). Its characteristics have been confirmed in both the demyelinating and myelin regeneration stages. For instance, CD11c^+^ microglia in the spinal dorsal horn have been found to be closely related to the repair of myelin after injury in the neuropathic pain model. This process involves their engulfing of myelin fragments and the release of the factor IGF-1 that promotes myelin regeneration (Kohno et al., 2022). In addition, resident microglia in the CNS are identified as the specific macrophage population that regulates myelin growth and integrity (McNamara et al., 2023). With the occurrence of myelin injury in stroke, the number of CD11c^+^ microglia significantly increases, and these cells exhibit efficient phagocytic ability and the expression of myelin support-related genes (Cao et al., 2021; Jia et al., 2023).

The functional state of CD11c^+^ microglia is regulated by multiple signaling pathways, with cytokines such as TGF-β and interleukin-10 influencing their activation level and functional characteristics (Zhang et al., 2023a). Intervention strategies aimed at promoting oligodendrocyte differentiation and myelin formation are receiving increasing attention. Strategies such as drug intervention, cytokine regulation, and gene therapy can effectively promote the maturation of oligodendrocytes and the formation of myelin. Ai et al. (2022) confirmed that natural components such as resveratrol and pomegranate extract can enhance myelin formation by activating the peroxisome proliferator-activated receptor gamma (PPARγ) signaling pathway.

Regulating the function of CD11c^+^ microglia is regarded as a new direction for intervening in neurodegenerative diseases. Clemastine is a first-generation antihistamine used to treat allergic symptoms and relieve itching (Yamazaki and Ohno, 2025). Mei et al. (2014) were the first to identify it as a candidate drug for demyelinating diseases, promoting the myelin regeneration of oligodendrocytes using a high-throughput screening platform of microcolumn arrays. However, despite an increase in the number of oligodendrocytes, clemastine reduced the conduction velocity of myelinated fibers in the corpus callosum during the developmental stage of mice (Palma et al., 2022). Subsequent studies have shown that this contradictory phenomenon may be related to the decreased activity of CD11c^+^ microglia and the reduced level of IGF-1 (Palma et al., 2022; Yamazaki and Ohno, 2025). It is suggested that clemastine may interfere with the signaling communication between microglia and oligodendrocytes (Palma et al., 2022). Therefore, while clemastine demonstrates a certain myelin repair effect, its mechanism of action is far more complex than initially observed. Further investigation is needed to address outstanding issues, such as optimizing the timing and duration of administration.

Gratimer acetate is often used in the treatment of solitary syndrome and relapsing-remitting MS in clinical practice. It is widely applied due to its high safety profile and minimal side effects. Koc et al. (2023) found that the prophylactic use of gratimer acetate in a mouse model of spontaneous photospinal encephalomyelitis could effectively delay disease progression. Its mechanism of action is related to CD11c^+^ cells. Experiments have shown that gratimer acetate exerts a protective effect by regulating the function of CD11c^+^ cells and reducing demyelinating lesions in the CNS (Koc et al., 2023).

The hedgehog and androgen signaling pathways functionally cooperate during developmental and repairing myelination (Shibuya et al., 2021). Shibuya et al. (2021) discovered the prominent myelin formation activity of testosterone. It may be involved in regulating the activity of microglia and astrocytes, maintaining the integrity of axon structures, inhibiting neuroinflammatory responses, and improving the prognosis of neurological function. Additionally, testosterone influences the peripheral immune system by promoting the expansion of tolerogenic CD11c^+^ cells, suppressing the clonal expansion of conventional CD4^+^ T cells, and increasing the population of immunosuppressive CD4^+^ FoxP3^+^ regulatory T cells (Shibuya et al., 2021).

Overall, a variety of drugs targeting the role of CD11c^+^ microglia in myelin repair have been proven effective at present. CD11c^+^ microglia may be used in the treatment of demyelinating diseases.

#### Regulation of neurogenesis and synaptic plasticity

In addition to supporting myelin regeneration, CD11c^+^ microglia can also promote neurogenesis and regulate synaptic plasticity. The complement system participates in neural development by clearing neuronal debris, phagocyte remnants, and defending against pathogens in a healthy brain. However, this system can become abnormally activated under pathological conditions, leading to the production of the allergic toxin C5a. The binding of C5a to C5aR1 triggers neuroinflammation and neuronal damage (Schartz and Tenner, 2020), making C5aR1 a key therapeutic target. In AD Arctic mice, C5aR1 knockout significantly slowed cognitive decline and protected neurons without affecting Aβ plaque accumulation (Hernandez et al., 2017). Blocking the C5a–C5aR1 interaction specifically delayed the expansion of CD11c^+^ microglia without altering other reactive microglia or astrocytes, indicating that this pathway primarily drives AD through microglial activation (Liddelow et al., 2017). While C5aR2 may offer neuroprotection, the interaction with C5aR1 is detrimental in AD (Carvalho et al., 2022), supporting the potential of C5aR1 inhibitors as promising therapeutics for AD.

The co-expression of *Itgax* and *Spp1* is significantly associated with specific subpopulations of CD11c^+^ microglia (Hammond et al., 2019; Li et al., 2019). Shen et al. (2022) discovered that OPN is mainly secreted by CD11c^+^ microglia, and its co-expression with CD11c marks a cell subpopulation with unique functions. This type of cell persists from the embryonic stage to adulthood, and its existence does not rely on external activation signals. The interaction between OPN and αVβ3 integrin receptors induces the production of TNF-αand activates the inflammasome, which may transform homeostatic microglia into a pro-inflammatory phenotype (Shen et al., 2022). Findings from Shen et al. (2022) have shown that using OPN-KO mice, the stable expression of CD11c in microglia may require the co-expression of OPN. These unique characteristics of the cell subpopulation provide important clues for understanding the differentiation mechanisms of microglia in both physiological states and disease processes. Qiu et al. (2023) crossbred 5XFAD transgenic mice with Spp1flstop (OPN-KO) mice and analyzed the effect of OPN on the pathology of AD using OPN-KO 5XFAD mice, confirming the absence of OPN at both the genetic and protein levels. It was demonstrated that OPN deficiency significantly reduced the pro-inflammatory response of microglia, decreased general and diffuse Aβ plaques, improved dystrophic neurites, and enhanced cognitive function in 5XFAD mice. Thus, OPN may be a promising therapeutic target for AD.

Scholars have revealed the potential harmful effects of CD11c^+^ microglia (Balasubramaniam et al., 2009; Qiu et al., 2023). For instance, CD11c has been confirmed as a marker for recognizing pro-inflammatory microglia subsets in retinal inflammation or injury models (Balasubramaniam et al., 2009). These cells inhibit the neurogenic ability of retinal progenitor cells by secreting inflammatory factors such as IL-6, thereby hindering the self-repair process of tissues. Activated microglia can migrate out of retinal explants, exhibiting typical pro-inflammatory phenotypic characteristics, including positive expression of inducible nitric oxide synthase and significant upregulation of immune-related markers such as CD45, CD11b, and CD11c. When stimulated by LPS and IFNγ, these CD11c^+^ cells secrete high levels of IL-6, significantly inhibiting the ability of retinal cells to form neurospheres. The presence of CD11c indicates that these cells are in an inflamed and activated state, potentially contributing to the suppression of the regenerative potential in endogenous retinal stem cells (Balasubramaniam et al., 2009). Therefore, regulating the active state of CD11c^+^ microglia or directly blocking the release of inflammatory mediators such as IL-6 may serve as an intervention strategy to alleviate retinal injury.

Skuljec et al. (2025) evaluated the role of CD11c^+^ microglia in the treatment of Alzheimer’s disease (AD) through T-cell immunotherapy using a double transgenic APP/PS1 mouse model. Experiments demonstrated that Aβ levels were significantly reduced, neuronal generation ability was enhanced, and the rate of cognitive decline was significantly delayed following specific vaccination regimens. The therapeutic effect was primarily attributed to the vaccine-induced shift in microglial polarization from a pro-inflammatory phenotype to an anti-inflammatory and reparative state.

Collectively, the role of CD11c^+^ microglia in neurogenesis and synaptic plasticity is expected to provide a promising direction for future treatment interventions.

#### Microenvironment reconstruction and neural repair

CD11c^+^ microglia are involved in microenvironment remodeling after brain injury, particularly in the thalamic region following a stroke (Deczkowska et al., 2018). The number of these cells increases significantly after ischemic events, exhibiting DAM-like characteristics, including the downregulation of homeostasis markers such as Tmem119 and Cx3cr1. Simultaneously, genes associated with inflammation and lipid processing—such as *Apoe*, *Axl*, *Lpl*, *Csf1*, and *Cst7*—are significantly upregulated (Cao et al., 2021). This phenotypic transformation suggests that CD11c^+^ microglia may actively participate in clearing lipid-rich debris from damaged neurons, modulating local inflammatory responses, and contributing to tissue remodeling. Notably, the activation of CD11c^+^ microglia can be observed within 24 hours after a stroke, occurring earlier than the evident neurodegenerative changes (Cao et al., 2021). This indicates that they may play a role in establishing the early inflammatory environment and influencing subsequent nerve injury processes. On one hand, they help eliminate cell debris and pathological proteins to maintain microenvironment stability. On the other hand, they secrete pro-inflammatory mediators and neurotrophic factors that can both inhibit and support neuronal vitality, while also participating in synaptic pruning and neural circuit adjustment. However, an overactivated state may exacerbate neuronal damage.

SIRPα is a membrane protein that is highly expressed in macrophages and microglia. It interacts with CD47 on neurons to inhibit microglial activation by generating a “Don’t eat me” signal (Li et al., 2025; Zhang et al., 2025a). Sato-Hashimoto et al. (2019) discovered CD11c^+^ microglia in the white matter of SIRPα-deficient mice, along with increased expression of innate immune molecules. Similar results were observed in CD47-deficient mice. Importantly, SIRPα deficiency alone does not induce white matter damage, indicating that the presence of CD11c^+^ microglia does not inherently reflect pathological inflammation but may instead represent an adaptive immunomodulatory response.

NMO is an autoimmune disease characterized by CNS damage (Holian and Weinshenker, 2025). Its core mechanism is autoantibody attack against aquaporin 4 (AQP4) (Abbasian et al., 2025; Guo et al., 2025). This kind of attack directly leads to astrocyte lesions and becomes a key pathological basis for disease development (Arzalluz-Luque et al., 2025; Lorefice et al., 2025). Traditional therapies (type I interferon (IFNI)-IFNβ), are ineffective and may exacerbate the disease (Wlodarczyk et al., 2021). Mice lacking IFNβ receptors exhibited a significant reduction in NMO-like pathology and a decrease in microglia activation. Treatment with IFNβ leads to deteriorating pathology and further activation of microglia, as demonstrated by the expansion of the CD11c^+^ subset of microglia (Wlodarczyk et al., 2021). Whether CD11c^+^ microglia facilitate progression of the disease, and whether they are induced as a response to IFNβ stimulation of phagocytosis of astrocytes by CD11c^+^ microglia, both require further investigation.

Miyake et al. (2025) treated experimental neuromyelitis optica spectrum disorder (NMOSD) mice with the anti-IL-6R antibody (MR16-1), resulting in an increased proportion of CD11c^+^ microglia in the spinal cord, along with upregulation of gene expression associated with phagocytic activity. The blockade of IL-6 signaling via IL-6R inhibition appears to modulate microglial polarization toward a CD11c^+^ phenotype, which is linked to enhanced phagocytic function in AQP4 peptide-immunized mice. This shift in microglial response was correlated with reduced disease severity, suggesting that IL-6R blockade may exert protective effects by promoting a more regulatory and tissue repair-associated microglial state (Miyake et al., 2025).

#### Microglia replacement therapy

Microglia exhibit an extraordinary capacity for regeneration, capable of repopulating the CNS within 1 week following near-complete depletion (> 99%) via pharmacological inhibition of CSF1R. This phenomenon is called microglia repopulation and represents the first recorded instance of large-scale cell regeneration in the CNS of adult mammals (Borjini et al., 2025; Chen et al., 2025a). Compared with resident microglia, repopulated microglia show differences in morphological and transcriptional profiles (Hafeez et al., 2025). The repopulated microglia demonstrate reduced branch complexity, shorter processes, and overall simplification of tree branches, even 28 days after repopulation. Transcriptomic analysis indicates that genes related to phagocytic activity (including *Clec7a*, *Itgax*, *Axl*, *Tyrobp*, and *Cst7*) and genes related to the inflammatory response (such as *Tlr12*, *Tnfaip3*, and *Ccrl2*) are significantly upregulated (Wickel et al., 2024). These changes may facilitate the transition from acute to chronic pain and potentially lead to pain resolution (Donovan et al., 2024). These findings suggest that the depletion and repopulation of microglia can drive the acquisition of the CD11c^+^ phenotype, which may help repair and regulate the pathological processes of neurological diseases.

Another microglia replacement strategy involves replacing genetically defective microglia with allogeneic healthy microglia. This method aims to restore normal immune surveillance, phagocytic activity, and neurosupport functions in the CNS, providing a potential treatment option for diseases caused by microglial deficiency (Rao and Peng, 2023). For diseases caused by microglial gene mutations, the ideal intervention approach is to use viral vectors to specifically deliver repair genes. Although vector-mediated gene therapy has been extensively investigated in preclinical models and some approaches have advanced to clinical trials (Bulaklak and Gersbach, 2020), microglia have shown significant resistance to viral vectors (Maes et al., 2019). This results in poor transduction efficiency or even complete failure of gene transfer, posing a significant challenge for the development of effective microglia-targeted gene therapies.

Given the limitations of both viral and non-viral gene delivery approaches, cell-based therapeutic strategies have gradually attracted attention. Bone marrow transplantation (BMT) has been shown to partially replace microglia in the CNS with donor-derived monocytes (Rao and Peng, 2023). However, preclinical and clinical studies have yielded suboptimal outcomes, primarily because the implantation efficiency of donor-derived microglia after BMT is relatively low, usually ranging from 2% to 20% (Rao and Peng, 2023). This limited repopulation capacity represents a major barrier to the therapeutic efficacy of traditional BMT strategies in targeting microglial dysfunction.

Mouse microglia cannot fully capture all aspects of human diseases. Fattorelli et al. (2021) directly transplanted microglia derived from human induced pluripotent stem cells or hematopoietic stem cells into the brains of recipient mice. However, this method has notable limitations. It requires the use of immunodeficient recipient mice (such as RAG2^–/–^ IL2Rg^–/–^ double knockouts) to prevent immune rejection of the transplanted cells. Another limitation is that the transplantation time window is restricted to the early postnatal period, when microglia have not fully occupied the brain and lack characteristic branching processes (Xu et al., 2020; Fattorelli et al., 2021). Therefore, there is an urgent need to develop a new strategy.

At present, a promising approach is to use CSF1R inhibitors (such as PLX5622, PLX3397, BLZ945 or GW2580) to perform pharmacological clearance on microglia, creating a vacant microglia niche within the CNS (Gerber et al., 2018). After the depletion of microglia, the recipient mice were subjected to radiation treatment before receiving traditional bone marrow transplantation. After the treatment with CSF1R inhibitors was discontinued, the host environment will gradually recover, thereby allowing donor-derived microglial cells to re-colonize and fill the entire CNS. This “depletion-repopulation” strategy significantly enhances the implantation efficiency of donor cells, providing a new approach for the treatment of microglia-related diseases.

Given the causal link between microglial genetic mutations and the development of neurological disorders, replacing dysfunctional microglia with healthy donor cells represents a promising therapeutic strategy for correcting microglial impairments. However, the long-term consequences and potential off-target effects associated with microglial replacement must be rigorously evaluated to ensure the safety and efficacy of such cell-based interventions (Yohei et al., 2022).

### Drug development strategies targeting CD11c^+^ microglia and safety evaluations

#### Potential as therapeutic target

CD11c^+^ microglia have emerged as a promising therapeutic target for neurological diseases characterized by neuroinflammation and neurodegeneration, which are major public health burdens marked by progressive neuronal loss and a decline in cognitive, motor, and behavioral functions (Menéndez and Manucha, 2023). Current therapeutic approaches offer limited efficacy, primarily halting disease progression rather than restoring lost neural functions. Given the unique functions of CD11c^+^ microglia, the development of therapeutic strategies targeting them is considered promising. Single-cell RNA sequencing (scRNA-seq) helps identify specific molecular markers characterizing the CD11c^+^ subgroup (Depp et al., 2025; Sankowski and Prinz, 2025). Additionally, proteomics and metabolomics have demonstrated their adaptive responses to pathological conditions, particularly in lipid metabolism and amino acid utilization, which are significantly relevant in APOE4-related neuroinflammation (Hollinger et al., 2020).

#### Pharmacological and immunotherapeutic approaches targeting CD11c^+^ microglia

A variety of therapeutic strategies targeting CD11c^+^ microglia are currently under investigation, spanning molecular, immunological, and cellular approaches. Gene-targeted interventions offer high specificity. Recent research by Gholampour et al. (2025) found that CD11c^+^ microglia exhibit higher inflammatory activity and metabolic reprogramming in AD, accompanied by the upregulation of genes such as *Neprilysin* and *Lamp2a*. Precise interventions targeting these genes will help specifically regulate CD11c^+^ microglia in AD.

Specific regulation of inflammatory signaling pathways is also regarded as a major therapeutic strategy. The C5a-C5aR1 axis is involved in driving microglial activation in AD. C5aR1 antagonists have shown efficacy in reducing Aβ plaque accumulation and improving cognitive function in mouse models (Carvalho et al., 2022). Similarly, IFN-I signaling contributes to the neuroinflammatory progression of NMOSD, where the blockade of IFNβ receptors weakens the activation and demyelinating responses of microglia. The application of anti-IL-6R antibodies (such as MR16-1) can enhance the phagocytic ability of CD11c^+^ microglia, thereby improving the pathology of NMOSD (Wlodarczyk et al., 2021). Targeting the PPARγ or SREBP pathways can shift energy metabolism from glycolysis to oxidative phosphorylation through metabolic regulation methods, restore microglial homeostasis, and inhibit pro-inflammatory responses (Ai et al., 2022).

Additionally, regulatory mechanisms targeting the immune system help the body recognize and eliminate foreign antigens, maintaining the stability of the internal environment. Kim et al. (2018) found that estrogen receptor beta receptor activation can down-regulate inducible nitric oxide synthase expression in CD11c^+^ microglia and enhance interleukin-10 secretion, demonstrating neuroprotective effects in EAE (Kim et al., 2018). The testosterone-activated Hedgehog signaling pathway supports oligodendrocyte differentiation and myelin regeneration, especially in MS models (Shibuya et al., 2021). The feasibility of CD11c^+^ microglia as a therapeutic target has been confirmed through gene editing techniques such as CRISPR and CRISPR-associated protein 9 conditional gene knockout experiments (Qiu et al., 2023). Additionally, induced pluripotent stem cell-derived microglia provide a platform for developing personalized cellular replacement therapies (Lopez-Lengowski et al., 2021).

#### Cellular crosstalk of CD11c^+^ microglia in neuroinflammation and repair

CD11c^+^ microglia play a role in the complex neuroimmune network and are involved in dynamic crosstalk that affects neurodegeneration and repair. In their interaction with neurons, microglia promote the transformation from CD11c^–^ to CD11c^+^ by phagocytosing apoptotic neurons in the early stages of development (Shen et al., 2022). Meanwhile, neonatal microglia exhibit unique myelin and neurogenic phenotypes, highly expressing Itgax and promoting myelination by secreting IGF-1 and interacting with oligodendrocytes (Wlodarczyk et al., 2017). While drugs such as clemastine may disrupt communication between microglia and OPCs, there is an increase in OPC numbers (Palma et al., 2022).

The presence of CD11c^+^ microglia in the periaqueductal area of the brain in 1-week-old mice is a new finding, especially in AQP4^–/–^ mice, where these cells persist for a relatively long time. This is significant because AQP4 is expressed on the foot processes of astrocytes, suggesting that these cells may interact with astrocytes to promote the normal development of the ependymal membrane (Mayo et al., 2024). The progression of EAE is associated with the reduction and dispersion of CD11c^+^ microglia along with parenchymal infiltration. The selective reduction of CD11c^+^ microglia in female mice with p38α knockout driven by the CD11c promoter is linked to an increased progression rate of EAE. In this context, CD11c^+^ microglia were found to come into contact with astrocytes at the glial boundary membrane, while immune cells remained in the perivascular space (Mayrhofer et al., 2021). This indicates that CD11c^+^ microglia may regulate the parenchymal infiltration of immune cells in autoimmune demyelination by interacting with astrocytes. Understanding these interactions is crucial for developing therapies that shift the balance from destructive inflammation to tissue repair.

#### Clinical barriers and future directions

Despite an increasing amount of preclinical evidence indicating that targeting microglia can potentially be applied in the treatment of CNS diseases, there are still many obstacles before these approaches can be translated into clinical applications. For instance, clemastine reduces the speed of nerve fiber transmission, although it promotes an increase in the number of oligodendrocytes. This might be related to the impaired interaction between CD11c^+^ microglia and oligodendrocytes (Palma et al., 2022). Furthermore, interferon-beta treatment for NMOSD may instead exacerbate neuroinflammation (Wlodarczyk et al., 2021). Additionally, PPARγ agonists restore microglia homeostasis through metabolic regulation but also carry the risk of inducing metabolic syndrome, highlighting the importance of developing selective regulators (Ai et al., 2022). Microglia replacement, combining genetic engineering with cell transplantation, is a cutting-edge approach for the precise treatment of microglia-related diseases but carries the risks of immune rejection and tumor formation (Zhang et al., 2023a).

Currently, preclinical studies have verified several promising intervention measures targeting CD11c^+^ microglia (Mayo et al., 2024; Miyake et al., 2025). However, further research is still needed to achieve true clinical transformation. This includes considerations of the permeability efficiency of the BBB, the heterogeneity of microglia subsets, and the need for individualized treatment. Notably, individuals carrying the APOE4 allele display unique microglial metabolic profiles, complicating the prediction of drug efficacy.

With the development of multi-omics and spatial transcriptomics technologies, the phenotypic characteristics and spatio-temporal distribution of CD11c^+^ microglia will be analyzed more precisely. Nanopharmacology is emerging as a new direction in the treatment of neuroinflammation. Newly developed nanoparticles can precisely target brain immune cells, such as microglia and astrocytes, effectively reducing neuroinflammation and protecting neurons. Nanoformulations targeting specific neurons have also been successfully developed, and related clinical trials are evaluating their safety and efficacy in the treatment of neurodegenerative diseases and mental disorders (Menéndez and Manucha, 2023).

### Current situation of clinical transformation

The transformation of CD11c^+^ microglia from the laboratory to clinical practice requires extensive testing and strict regulatory approval processes. At the regulatory level, various countries adopt different strategies to promote drug development. The successful advancement of CD11c^+^ microglia-targeted therapies necessitates strategic alignment with local regulatory expectations. Clinical trials targeting microglia are currently underway, including novel therapies such as TREM2 agonist antibodies, CD33 antagonist antibodies, RIPK1 inhibitors, and P2X7 receptor antagonists (Ling and Crotti, 2024; **Box 2**).

Box 2: Progress in clinical translation of the researches targeting microglia

**1. Global distribution of clinical trials**

(1) At present, clinical trials targeting microglia are unevenly distributed globally, mainly concentrated in regions such as North America, Europe, and Australia. Furthermore, most of these trials involved targets such as TREM2 (Long et al., 2024), CSF1R (https://clinicaltrials.gov/study/NCT04121208), CD33 (Ling and Crotti, 2024), RIPK1 (Vissers et al., 2022), and P2X7 (Timmers et al., 2018; Recourt et al., 2020, 2023). It mainly targets diseases such as Alzheimer’s disease (NCT04592874, NCT03635047), amyotrophic lateral sclerosis (NCT03757351), mild cognitive impairment (NCT04121208), major depressive disorder (NCT02902601), drug-resistant hypertension (NCT02213575) and regressive autism (NCT00409747). Among them, most are Phase II trials, mainly focusing on evaluating safety and preliminary efficacy, which indicates that the treatment targeting microglia has a very good application prospect.

(2) The number of clinical trials related to microglia registered on ClinicalTrials.gov has been increasing every year, and has also received support from Alector, Inc., University of Oxford, Denali Therapeutics Inc., Sanofi and Janssen Research & Development.

**2. U.S. Food and Drug Administration approval**

(1) Some drugs targeting microglia have received conditional approval from the U.S. Food and Drug Administration, mainly for delaying the progression of Alzheimer’s disease (NCT03635047, NCT04592874). Their mechanism of action is mainly to have good dose tolerance and targeting, and to produce neuroprotective effects, without significant treatment-related adverse events (Ma et al., 2025). Therefore, the approval of these drugs by the U.S. Food and Drug Administration marks a crucial step forward in the clinical application of related technologies for microglial therapy.

(2) In the past few years, the number of drug patent applications in the field of microglia has shown a significant upward trend, reflecting the emphasis on targeted microglial cell therapy. The development of therapeutic compounds in the clinical stage to improve microglial function can serve as a transformative approach to alleviate neurological disorders. Enhancing our ability to promote the homeostasis and repair functions of microglia is expected to bring better outcomes for patients affected by neurological diseases.

**3. Financial support from the National Institutes of Health**

(1) The financial support from the National Institutes of Health of the United States for clinical research on microglia has not yet been demonstrated. However, considering the beneficial results obtained from these preclinical studies and clinical trials, research in these fields is likely to receive strong support from the National Institutes of Health.

(2) Research and application of microglial therapy in the field of nervous system repair have shown significant potential for clinical transformation. The number of clinical trials in this field has increased significantly, especially those related to Alzheimer’s disease (Ma et al., 2025). This indicates that the medical community’s interest and expectations in this field are on the rise.

TREM2 is an immune receptor expressed exclusively in the brain’s microglia, and its loss of function is associated with an increased risk of AD (Jung et al., 2025). Enhancing TREM2 function may help attenuate the pro-inflammatory response of microglia in AD. Multiple clinical trials have been conducted for treatments targeting TREM2. Alector Inc. has collaborated with AbbVie to develop an agonist monoclonal antibody named AL002, which aims to enhance TREM2 signaling by preventing the lysis and shedding of the TREM2 protein from the cell membrane. This promotes continuous surface expression and activation of microglial cell function. Preclinical studies have shown that AL002 enhances microglial activation and phagocytic activity in the 5XFAD mouse model, reduces the burden of amyloid plaques and synaptic pathology, and improves cognitive behavioral deficits (Wang et al., 2020). A Phase I clinical trial (ClinicalTrials.gov/NCT03635047, 2018) demonstrated the safety and pharmacodynamic activity of AL002 in healthy volunteers, showing a dose-dependent reduction of sTREM2 and an increase of sCSF1R in cerebrospinal fluid (CSF). Meanwhile, AL002 induced microglial proliferation (Long et al., 2024). These findings support further clinical development, and a Phase II trial (ClinicalTrials.gov/NCT04592874, 2020) is currently underway to evaluate the safety and efficacy of AL002 in patients with early-stage AD, with the primary endpoint being the change in the Clinical Dementia Rating Sum of Boxes (CDR-SB) score.

In parallel, another therapeutic drug targeting TREM2 called DNL919, is being developed by Denali Therapeutics in collaboration with Takeda. Kariolis et al. (2020) utilized antibody transport vectors to enhance the penetration of the BBB. The immunogenic IgG mouse substitute 4D9 was detected in APP-NL-G-F knock-in mice to induce microglia to transition from homeostasis to DAM. It was manifested as a decrease in P2Y12 and an increase in TREM2 staining in Aβ plaques around microglia (Schlepckow et al., 2020). DNL919 is currently in Investigational New Drug (IND)-enabling studies (Ling and Crotti, 2024), marking a key step toward clinical evaluation.

hT2AB is an investigational therapeutic drug developed by Amgen that aims to inhibit the shedding of TREM2 by specifically targeting the stem region of the receptor. A preclinical study conducted in TREM2 variant (CV/R47H) 5XFAD mice demonstrated that hT2AB promotes microglial proliferation and enhances the secretion of pro-inflammatory cytokines (Ellwanger et al., 2021). hT2AB has been acquired by Vigil Neurosciences and is currently advancing through IND development.

CSF1R is a tyrosine kinase transmembrane receptor and a member of the growth factor CSF1/platelet-derived growth factor receptor family, exhibiting inherent tyrosine-specific protein kinase activity. CSF1R is involved in the survival, proliferation, differentiation, recruitment, and function of mononuclear phagocytes (macrophages, monocytes, and microglia) (Hammond et al., 2025; Lee et al., 2025). Currently, there are three approved CSF1R inhibitors: Pexidartinib, Nilotinib, and Surufatinib, which are primarily used for tumor treatment. The development and clinical evaluation of therapeutic drugs targeting CSF1R for neurological diseases are also gradually advancing. Preclinical studies have demonstrated that Edicotinib suppresses microglial proliferation, reduces the production of pro-inflammatory cytokines, including IL-1β and TNFα, decreases tau phosphorylation, and ameliorates neurodegeneration and motor dysfunction (Mancuso et al., 2019; Vicente-Rodríguez et al., 2023). In collaboration with the University of Oxford, Janssen Pharmaceuticals initiated a Phase I clinical trial (ClinicalTrials.gov/NCT04121208, 2019) to assess the safety and tolerability of Edicotinib in patients with mild cognitive impairment. A key objective of this study was to identify potential biomarkers predictive of therapeutic response, thereby informing the design and endpoints of future Phase II trials aimed at evaluating its disease-modifying potential in early-stage neurodegenerative conditions.

CD33 is an immune receptor that inhibits the activity of microglia and plays a negative regulatory role in AD (Beckers et al., 2024). A humanized monoclonal antibody AL003 targeting CD33 has entered the clinical trial stage and has shown good therapeutic potential in an early study (Ling and Crotti, 2024). AL003 can reduce siglec3 expression, resulting in increased activated microglia in the brain (ClinicalTrials.gov/NCT03822208 2019). Clinical data have shown that AL003 is well tolerated and exhibits target engagement in both peripheral blood and CNS. To further evaluate its efficacy in early-stage AD, a randomized, placebo-controlled Phase II clinical trial is planned to assess the drug’s disease-modifying effects in a larger patient population.

Receptor-interacting serine/threonine protein kinase 1 (RIPK1) is a key signaling protein that exerts pro-inflammatory and pro-death signaling effects after tumor necrosis factor (TNF) activates its receptor TNFR1 (Chen et al., 2025b). RIPK1 not only acts as a scaffold protein in the NF-κB pathway to regulate cell survival (Eldesoqui et al., 2025), but it can also induce apoptosis or necroptosis through its kinase activity, especially in the complex IIb formed by RIPK1, RIPK3, and MLKL (Wagner et al., 2025; Zhang et al., 2025c). Studies have shown that abnormal activation of RIPK1 is closely related to neuroinflammation and neuronal death in various neurodegenerative diseases, including AD, ALS, and MS (Lu et al., 2025; Johnson and Lukens, 2025). To target this pathway, Denali Therapeutics has developed the brain-penetrating RIPK1 inhibitor DNL747. Clinical trials were conducted for ALS patients (ClinicalTrials.gov/NCT03757351, 2018) and AD patients (ClinicalTrials.gov/NCT03757325, 2018). DNL747 demonstrated good targeting and tolerance in early clinical trials, but toxicity at high doses was found in an animal study, which limited its further development (Grievink et al., 2019). Currently, its new-generation candidate drug, DNL788, has demonstrated a superior therapeutic window and has shown good target binding ability in healthy volunteers. It is planned to enter Phase II clinical trials for the treatment of ALS and has been granted Fast Track designation by the FDA.

ATP is an important signaling molecule released by neurons and glial cells, which activates microglia as a danger signal under pathological conditions such as tissue damage or inflammation (Ren et al., 2025; Yi et al., 2025). Microglia sense high concentrations of ATP through P2X receptors (especially P2X7), thereby triggering the release of pro-inflammatory factors including IL-1β, and are involved in the pathogenesis of neurodegenerative and mood disorders (Schäfer et al., 2022). Studies have shown that P2X7 is upregulated in diseases such as neuropathic pain, AD, and depression (Alves et al., 2025; Shen et al., 2025). Inhibiting this receptor can reduce inflammatory responses and improve behavioral symptoms (Bi et al., 2025; Shen et al., 2025). Janssen Pharmaceuticals is developing CNS-permeable P2X7 antagonists, including JNJ-54175446, and has conducted several clinical trials (ClinicalTrials.gov/NCT03088644, 2017; ClinicalTrials.gov/NCT03058419, 2017; ClinicalTrials.gov/NCT02930694, 2016; ClinicalTrials.gov/NCT02933762, 2016; ClinicalTrials.gov/NCT02902601, 2016). These trials demonstrated good safety, brain penetration ability, and potential effects on inflammation and mood regulation in Phase I clinical trials (Recourt et al., 2020). The project is currently in Phase II trials to assess efficacy in patients with major depressive disorder (ClinicalTrials.gov/NCT04116606, 2019).

This section summarizes the research progress and existing challenges in the treatment of neurodegenerative diseases and neuropsychiatric disorders involving microglia. Currently, a variety of therapies have either entered or are about to begin clinical trials, with the aim of improving outcomes for patients affected by these diseases.

## Limitations

First, although CD11c^+^ microglia have been described in multiple studies, there is currently no unified definition or standard. Many factors induce the generation of this subgroup, and the marker genes and thresholds used in different studies vary greatly. Second, most existing evidence relies on correlational analyses, lacking specific genetic tools to manipulate this cell population and validate causal mechanisms. Third, current research is primarily based on animal models, which exhibit significant differences from human genetics, resulting in limited cross-species validation. Finally, limitations in targeted delivery technologies and preclinical modeling pose significant barriers to therapeutic translation. Therefore, further in-depth investigation into the mechanisms associated with CD11c^+^ microglia—particularly focusing on precise regulatory strategies and clinical applicability—is critically needed.

## Conclusion

As a disease-associated, functionally diverse subset of microglia, CD11c^+^ microglia represent a promising frontier in neuroimmunology. This review highlights their scientific significance in bridging neuroinflammation and neural repair through dynamic phenotypic plasticity, regulated by evolutionarily conserved pathways such as SIRPα-CD47, complement activation, and IGF-1 signaling. Compared with earlier reviews, our work offers an innovative, cross-disease perspective that reveals common regulatory principles of CD11c^+^ microglia across AD, PD, ALS, MS, and brain injury, demonstrating their universality as both pathological and reparative cell states. The practical and clinical significance of these findings lies in their potential to guide the development of precision immunomodulatory therapies.

## Data Availability

*Not applicable.*
